# Supplementary material for: Resolving the full spectrum of human genome variation using Linked-Reads
Source: Genome Res. 2019 Apr;29(4):635–45. doi: 10.1101/gr.234443.118 (PMC6442396; doi:10.1101/gr.234443.118)
Supplement: Supplemental Material [file supp_gr.234443.118_Supplemental_longranger-2.2.zip › longranger-2.2.tar.bz2!/lr2.2-public/lib/stan/cmdstan/stan/doc/api/html/fwd_2functions_2hypot_8hpp_source.html]

Stan: src/stan/agrad/fwd/functions/hypot.hpp Source File


|  |  |
| --- | --- |
|  | Stan  2.5.0  probability, sampling & optimization |


- Main Page
- Related Pages
- Namespaces
- Classes
- Files

- File List
- File Members

All Classes Namespaces Files Functions Variables Typedefs Enumerations Enumerator Friends Macros Pages

- src
- stan
- agrad
- fwd
- functions

hypot.hpp

Go to the documentation of this file.

1 #ifndef STAN\_\_AGRAD\_\_FWD\_\_FUNCTIONS\_\_HYPOT\_HPP

2 #define STAN\_\_AGRAD\_\_FWD\_\_FUNCTIONS\_\_HYPOT\_HPP

3

4 #include <stan/agrad/fwd/fvar.hpp>

5 #include <stan/meta/traits.hpp>

6 #include <stan/math/functions/inv.hpp>

7 #include <math.h>

8

9 namespace stan {

10

11  namespace agrad {

12

13  template <typename T>

14  inline

15  fvar<T>

16  hypot(const fvar<T>& x1, const fvar<T>& x2) {

17  using ::hypot;

18  using ::sqrt;

19  using stan::math::inv;

20  T u = hypot(x1.val\_, x2.val\_);

21  return fvar<T>(u, (x1.d\_ \* x1.val\_ + x2.d\_ \* x2.val\_) \* inv(u));

22  }

23

24  template <typename T>

25  inline

26  fvar<T>

27  hypot(const fvar<T>& x1, const double x2) {

28  using ::hypot;

29  using ::sqrt;

30  using stan::math::inv;

31  T u = hypot(x1.val\_, x2);

32  return fvar<T>(u, (x1.d\_ \* x1.val\_) \* inv(u));

33  }

34

35  template <typename T>

36  inline

37  fvar<T>

38  hypot(const double x1, const fvar<T>& x2) {

39  using ::hypot;

40  using ::sqrt;

41  using stan::math::inv;

42  T u = hypot(x1, x2.val\_);

43  return fvar<T>(u, (x2.d\_ \* x2.val\_) \* inv(u));

44  }

45

46  }

47 }

48 #endif

stan::math::inv

boost::math::tools::promote\_args< T >::type inv(const T x)

**Definition:** inv.hpp:12

stan::agrad::hypot

var hypot(const double &a, const stan::agrad::var &b)

Returns the length of the hypoteneuse of a right triangle with sides of the specified lengths (C99)...

**Definition:** hypot.hpp:119

stan::agrad::fvar

**Definition:** fvar.hpp:13

stan::agrad::hypot

fvar< T > hypot(const fvar< T > &x1, const fvar< T > &x2)

**Definition:** hypot.hpp:16

traits.hpp

stan::agrad::inv

fvar< T > inv(const fvar< T > &x)

**Definition:** inv.hpp:15

inv.hpp

stan::agrad::sqrt

fvar< T > sqrt(const fvar< T > &x)

**Definition:** sqrt.hpp:15

fvar.hpp

stan::agrad::fvar::d\_

T d\_

**Definition:** fvar.hpp:16

stan::agrad::fvar::val\_

T val\_

**Definition:** fvar.hpp:15


---

[ Stan Home Page ]

*© 2011–2014,
Stan Development Team.*
